# Teething disturbances; prevalence of objective manifestations in children under age 4 months to 36 months

**DOI:** 10.4317/medoral.17487

**Published:** 2011-12-06

**Authors:** Roshan Noor-Mohammed, Sakeenabi Basha

**Affiliations:** 1 MDS Reader, Department of Pedodontics and preventive dentistry, College of dental sciences, Davangere, Karnataka, India, 577004; 2 MDS Reader, Department of Community Dentistry, College of dental sciences, Davangere, Karnataka, India, 577004; 3 ….

## Abstract

Objective: The aim of this study was to present data as responded by parents on teething manifestation during eruption of primary teeth and the occurrence of objective manifestations in children ages 4 months to 36 months. Settings and Design: Hospital based face-to-face questionnaire study. Study Design: One thousand and one hundred children ages four to 36 months who had at least one erupting tooth were included in the study. Parents were asked to complete a short questionnaire and children were then checked by one of the authors. Statistical analysis used: Chi-square analysis was performed to analyze information obtained. Level of significance was set at P<.05. Results: There were 660 girls (60%) and 440 boys (40%) in the study. The most frequent clinical manifestations were: Fever (16%), drooling (12%), diarrhea (8%), fever-drooling (15%), fever-diarrhea(8%) and drooling-diarrhea (6%). In the study sample, boys demonstrated a higher prevalence of diarrhea than girls (P<.05). No statistical significance regarding other clinical manifestations and gender were observed. Teething manifestations were most prevalent during the eruption of primary incisors. Occurrence of clinical manifestations in 4-12months and 13-24 months age was statistically significant when compared with 25-36months age (P<0.05). Conclusions: An association has been shown between general objective manifestations like fever (the most prevalent), drooling and diarrhea, and the eruption of primary teeth. Most manifestations appeared during the eruption of the primary incisors.

** Key words:**Teething, primary teeth, eruption.

## Introduction

Teething is a natural physiological process that usually occurs without problems. It consists of the migration of the tooth from its intraosseous position in the jaw to eruption in the oral cavity (1).

Some authors have associated primary tooth eruption with alterations such as irritability, gingival irritation, increased salivation, fever, agitated sleep, diarrhea, and loss of appetite (2,3). These disturbances are responsible for the referral of many babies to dental practitioners, since they provoke discomfort and pain in the patient. Parents always ask about the probable relationship between these phenomena and the eruption of the primary teeth.

The relationship between tooth eruption and organic or systemic manifestations in children is controversial among dentists and physicians within the literature (4). It remains unclear whether the disturbances are caused by the eruption of the primary teeth or whether they simply coincide with tooth eruption. Since these disturbances are mainly observed during the eruption of the primary teeth, the objective of the present study was to determine their occurrence in a population seen at a child health institute and research center.

## Material and Methods

The study was conducted in the Child Health institute and research center. Children who visited the child health institute over the period of six months were examined and one thousand and one hundred children were selected who satisfied following inclusion criteria,

1. Children age between four months to 36 months

2. Displayed at least one tooth in the process of eruption. Eruption was determined if the clinical crown of the tooth was visible, but not exceeding 3 mm exposure above the gingival.

Written consent was procured from all the parents who participated in the study. Ethical clearance for the study was procured from the ethical committee of the institution prior to the study. Data was obtained mostly from the mothers who were the accompanying parent most of the time. Parents were asked to complete a short and simple questionnaire in local language. Information was relayed in a yes/no manner about three objective manifestations noted during the eruption of the primary incisors, canines, and molars, including drooling, diarrhea, fever, and the combination of these symptoms. Drooling was defined as excessive saliva coming out of the mouth like bubbles or continuous salivation. Oral examination of the child was done by one of the authors. Data was analyzed using descriptive statistics. Chi-square analysis was performed for the information obtained. Level of significance was set at P<.05.

## Results

There were 660 (60%) girls and 440 boys (40%) in the sample selected. ( Table 1) shows the distribution of the clinical manifestations that were registered in the study. In 32% of the children, no clinical manifestations were noted. In 68% of children one or more of the symptoms were registered. Each manifestation appeared alone or in combination with others. The most frequent clinical manifestations were: fever (16%), drooling (12%), diarrhea (8%), fever-drooling (15%), fever-diarrhea (8%), drooling-diarrhea (6%) and the combination of fever-drooling-diarrhea was found in 3% of the children.

**Table 1:**
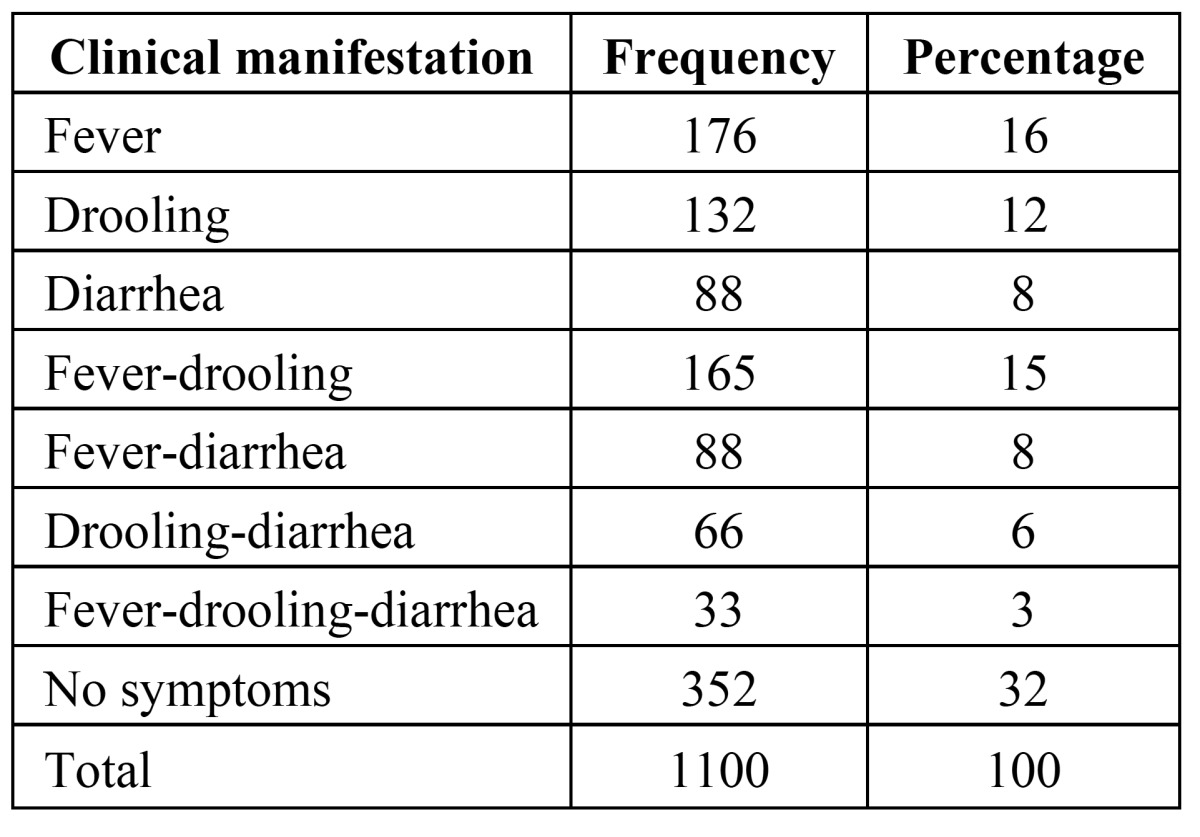
Distribution of the Clinical Manifestations in the Study Population.

In the study, boys demonstrated a significantly higher prevalence of diarrhea than girls (P<.05). No statistical significance regarding other clinical manifestation and gender were observed. ( Table 2) shows the clinical manifestations that were present during the eruption of the primary teeth according to the different type of teeth. Individually fever was significantly more prevalent during eruption of the incisors. ( Table 3) shows the frequency of clinical manifestations according to age. Most clinical manifestations were observed between the ages of 4 to 24 months. Mean age for reporting of first objective sign of teething was 7 ½ months. The clinical manifestations decreased with age. Fever was the most frequent clinical manifestation followed by drooling and fever-drooling between the ages of 4 to 12 months. Fever, drooling and fever-drooling were more prevalent objective signs in study sample respectively. Regarding the occurrence of clinical manifestations in 4-12 months and 13-24 months age group there was no statistical significance (P>0.05). There was statistically significant difference on comparison of clinical manifestations between 4-12 months group with 25-36 months age group (P<0.05) and 13-24 months group with 25-36 months group children (P<0.05).

**Table 2:**
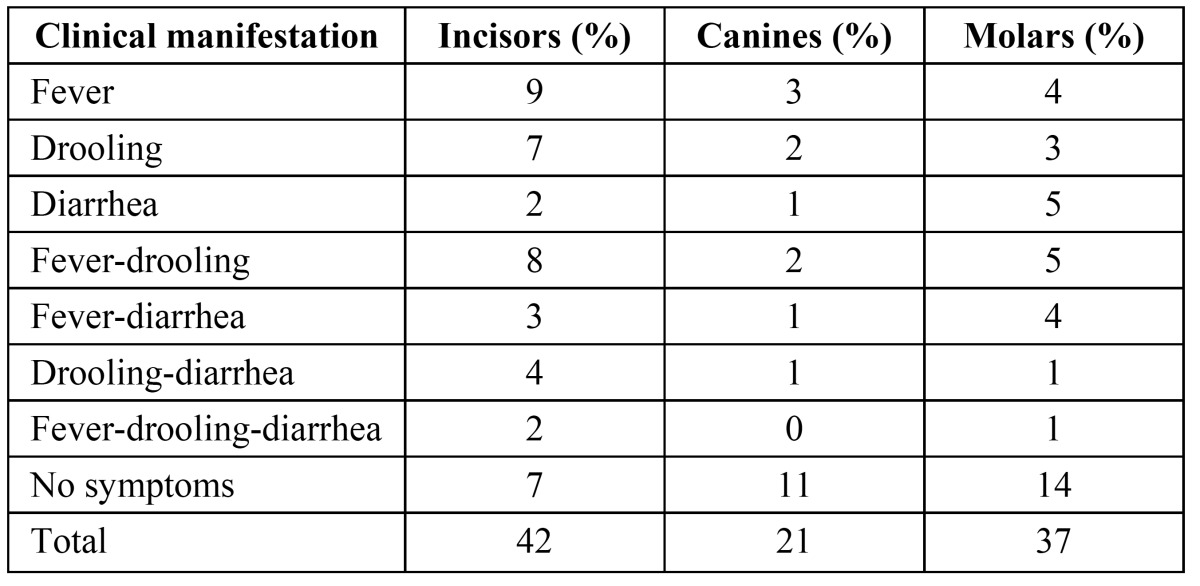
Prevalence of Clinical Manifestations According to Tooth-Type.

**Table 3:**
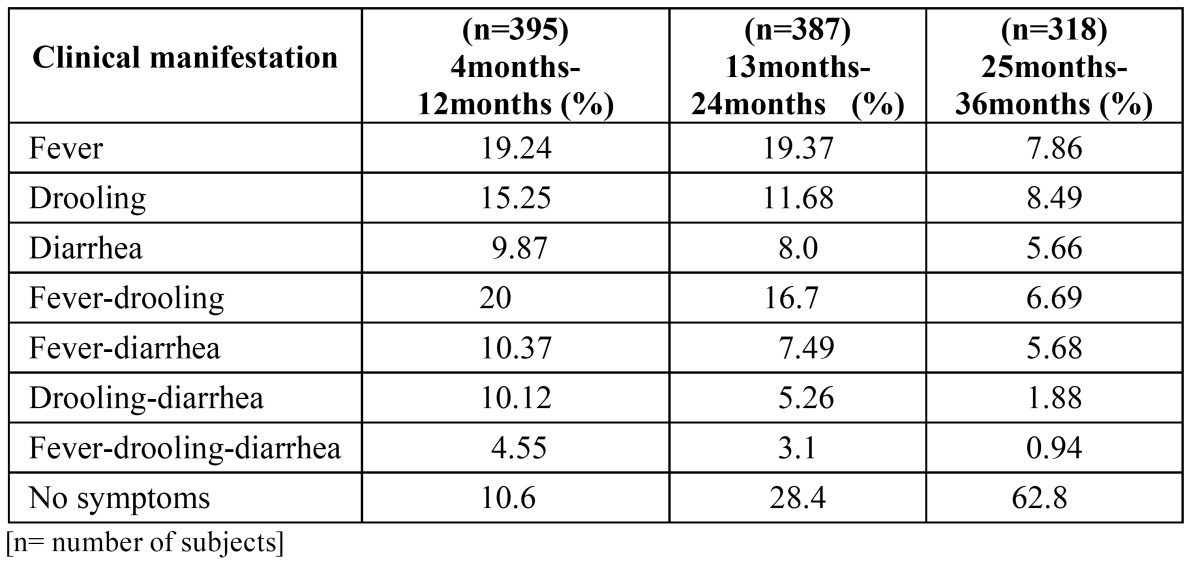
Prevalence of Clinical Manifestations According To Age.

## Discussion

The studies on teething disturbances in children have reported teething symptoms in as high as 80-90% of their study population (5), the results of this study showed that 68% of children with erupting teeth demonstrated general systemic symptoms like drooling, diarrhea, and fever, whereas 32% of study sample was asymptomatic. These symptoms could appear alone or in a combination with others. Lower percentage of teething symptoms in this study may be due to not considering symptoms such as irritability, gingival irritation and disturbed sleep which are subjective in nature. The findings were in accordance with the results of previous studies (6-11). The presence of fever alone and in combination of other symptoms was 16% and 26% respectively. On the whole fever reported in this study either alone or in combination was in high percentage of children (42%) which is in accordance with the findings of GaliIi et al. (8), Carpenter (7), and Jaber et al. (9) who also showed a high percentage of children with fever. Multiple tooth eruptions may establish a stress condition, during which the resistance against infections is reduced and incidence of infectious diseases is increased. Bennet and Brudno (12) suggest that fever during the process of primary tooth eruption is caused by the human teething virus (HT virus), which, at the beginning of life, is responsible for a primary infection that becomes subclinical. The HT virus remains in a latent state in the alveolar crypt until its stimulation through eruptive movements, provoking fever as well as local signs and symptoms such as gingival inflammation, hemorrhage, and pain (12).

This study showed that fever and drooling separately and in combination were the most common manifestations accompanying the eruption of the incisors. Drooling may be explained by the fact that the child agitates the oral cavity, producing irritation and redness of the gums (13).

The authors found that the clinical manifestations associated with the teething process decreased with age. Most symptoms were found between the ages of 4 to 24 months, while fever, the most common manifestation, was most prevalent between the ages of 4 to 12 months. The authors could find no explanation concerning the finding that boys demonstrated significantly higher prevalence of diarrhea in the children under study. Foster and Hamilton (14) have suggested that diarrhea during tooth eruption is associated with the contamination of the baby’s fingers or objects put into the mouth. There was statistically significant result on comparison between 4-12 months with 25-65 months age group suggesting increased occurrence of clinical manifestations during 4-12 months age. Occurrence of clinical manifestations was not statistically significant between 4-12 month and 13-24 months. The dental and pediatric literature presented different opinions regarding general symptoms related to children’s teething, which were not always data-based and were contradictory. The subjective nature of the information provided by the parents was one of the reasons for this. It is difficult to separate the signs and symptoms related to dental eruption from changes in the behavior of the child based solely on the parents’ subjective views. This was due to the extended period of time of the teething. Drooling at the age of 4 or 5 months could have been associated with dental eruption, but it also could have been a sign of the normal activity of the salivary glands (15-17).

Despite the fact that there was an agreement about the presence of symptoms during the eruption of primary teeth, some authors totally objected to a cause-effect association between them (17). It should be remembered that coincidentally, primary tooth eruption begins when infants lose maternal antibody protection against bacteria and viruses; making the baby more vulnerable to general threatening conditions as the newly pierced gingiva around an erupting tooth offers a convenient viral infection site (18).

Focusing on the objective signs allowed the authors to overcome a possible bias that could have been present if the data were obtained solely from the parents, or if other subjective symptoms would have been studied and gleaned some light on this somewhat unsolved issue.

The study was limited by the study sample, which were selected from the people visiting one particular child health institute. This of course, may not be representative of whole of the population. Also, only three signs were examined in the study: drooling, diarrhea and fever. Further research is needed on larger populations and should include more signs. It should be noted, however, that before signs or symptoms of a potentially serious illness can be attributed to infants’ teething, other possible causes must be ruled out. On the basis of the results of this study, the authors arrived at the following conclusions: a) An association has been shown between general objective signs (drooling, fever, and diarrhea) and the eruption of primary teeth with fever being the most prevalent sign followed by drooling and fever-drooling combination. b) Most signs appeared during the eruption of the primary incisors. c) When an infant at teething age has some symptoms, they may be attributed to teething but other possible causes must be ruled out first.
